# Brain region networks for the assimilation of new associative memory into a schema

**DOI:** 10.1186/s13041-022-00908-9

**Published:** 2022-03-24

**Authors:** Tomonori Takeuchi, Makoto Tamura, Dorothy Tse, Yasushi Kajii, Guillén Fernández, Richard G. M. Morris

**Affiliations:** 1 Centre for Discovery Brain Sciences, Edinburgh Neuroscience, University of Edinburgh, 1 George Square, Edinburgh, EH8 9JZ UK; 2 Danish Research Institute of Translational Neuroscience, DANDRITE, Nordic-EMBL Partnership for Molecular Medicine, Department of Biomedicine, Aarhus University, Hoegh-Guldbergsgade 10, 8000 Aarhus C, Denmark; 3 Center for Proteins in Memory, PROMEMO, Danish National Research Foundation, Department of Biomedicine, Aarhus University, Hoegh-Guldbergsgade 10, 8000 Aarhus C, Denmark; 4 Neuroscience Research Unit, Mitsubishi Tanabe Pharma Corporation, Kanagawa, 227-0033 Japan; 5 Department of Psychology, Edge Hill University, Ormskirk, L39 4QP UK; 6 T-CiRA Discovery, Takeda Pharmaceutical Company Limited, Kanagawa, 251-8555 Japan; 7 Donders Institute for Brain, Cognition and Behaviour, Radboud University Medical Center, Nijmegen, 6500 HB The Netherlands; 8 NeuroDiscovery Lab, Mitsubishi Tanabe Pharma Holdings America, Cambridge, MA 02139 USA

**Keywords:** Schema, Memory assimilation, Paired-associate memory, Functional network, Prelimbic cortex, Anterior cingulate cortex, Anterior retrosplenial cortex, Hippocampus

## Abstract

Alterations in long-range functional connectivity between distinct brain regions are thought to contribute to the encoding of memory. However, little is known about how the activation of an existing network of neocortical and hippocampal regions might support the assimilation of relevant new information into the preexisting knowledge structure or ‘schema’. Using functional mapping for expression of plasticity-related immediate early gene products, we sought to identify the long-range functional network of paired-associate memory, and the encoding and assimilation of relevant new paired-associates. Correlational and clustering analyses for expression of immediate early gene products revealed that midline neocortical-hippocampal connectivity is strongly associated with successful memory encoding of new paired-associates against the backdrop of the schema, compared to both (1) unsuccessful memory encoding of new paired-associates that are not relevant to the schema, and (2) the mere retrieval of the previously learned schema. These findings suggest that the certain midline neocortical and hippocampal networks support the assimilation of newly encoded associative memories into a relevant schema.

**Supplementary Information:**

The online version contains supplementary material available at 10.1186/s13041-022-00908-9.

## Introduction

Knowledge consisting of past experiences and facts stored in long-term memory is thought to be stored within anatomically distributed neuronal networks of cortical, allocortical and subcortical brain areas, an idea dating back to Hebb’s concept of ‘cell-assemblies’ [1]. The formation of long-term memories in vertebrates is mediated, in part, by activity-dependent changes in the strength of connections between neurons in the brain [1–4]. An initial trigger, at the time of the event, sets in motion a series of cellular and molecular changes within and across brain cells that give rise to the creation of a distributed ‘trace’ or ‘engram’ that outlasts the triggering event. Subject to a process called initial or ‘cellular’ consolidation, this can result in longer lasting changes in synaptic strength within brain structures such as the hippocampal formation [5, 6]. This initial process of memory persistence may then be accompanied or followed by a separate ‘systems’ consolidation process that somehow gives rise to lasting structural changes in neuronal connectivity in the neocortex [7–9]. Later neural activity can then activate relevant synapses to re-evoke activity patterns within these distributed networks that underlie retrieval of memory.

Distinct brain structures are important for different forms of memory with, for example, the hippocampal formation critical for the formation of declarative memory (episodic, spatial and semantic), the amygdala for emotional memory, and other structures for habit formation [10]. However, these and other brain structures are thought to encode information with regions of the neocortex [11, 12] and in parallel with neocortical regions during initial memory encoding [13–18]. If systems consolidation is activated, interaction between traces in these separate brain areas can lead to further changes in the neocortical network and so stabilize lasting memory. This 'parallel' framework differs from the sometimes stated textbook idea that memory traces are transferred between brain areas in sequence. However, direct evidence for these interacting network changes is lacking.

It has been challenging to define which subsets of neurons are parts of a distributed engram for two reasons [19]. First, the distributed nature of memory traces indicates that neurons in several brain regions will likely operate in a collective manner that may change with the passage of time. Second, it may not be the overall level of neural activity that is important but the spatiotemporal activation of cell-assemblies when memories are formed or retrieved. Using elegant immunocytochemical and statistical techniques, Wheeler and colleagues [19] showed how distributed patterns of expression of the immediate early gene (IEG) *Fos* protein products at the time of memory retrieval can shed light on time-dependent changes in connectivity throughout the brain over the course of systems memory consolidation. Their findings indicate that the cortical network activated at the time of retrieval a long time after context fear conditioning differs strikingly from that activated at the time of retrieval soon after learning. In a follow-up study, 21 different brain regions in mice were chemogenetically silenced one by one immediately after contextual fear conditioning and tested 10 days later [20]. It was observed that the degree of impaired memory consolidation and retrieval was correlated with the degree to which the brain region being silenced is functionally connected with other regions in the fear memory network.

Our project was conducted independently and focused on memory *encoding* rather than *retrieval.* Studies of the phenomenon of memory reconsolidation, in which memory retrieval conducted in the presence of a mRNA translation inhibitor (e.g. anisomycin) can sometimes result in the de-stabilization of memory, indicates that retrieval can be associated with gene activation [21]. In memory encoding, on the other hand, post-translational steps (such as synaptic potentiation) are a likely first step of regulating variety of cellular processes leading to the formation of a lasting memory trace [4] alongside gene activation in anticipation of other later events [22, 23]. Accordingly, we adopted similar methods to investigate the patterns of IEG product expression to those of [19] but in association with memory formation (encoding). Moreover, as our focus was less on de novo memory formation using un-trained animals but the assimilation of new information into existing networks, our studies were conducted using trained animals with previously acquired knowledge. Rather than use *Fos*, which is activated by neural activity [24], we monitored early growth response protein 1 (*Egr1*) and activity-regulated cytoskeleton-associated protein (*Arc*) that are activated in association with synaptic plasticity [25, 26]. Given the strong association between synaptic plasticity and memory formation, these IEGs are likely to be expressed more selectively at the time of, or soon after, memory encoding. Instead of investigating memory for the context of a fearful experience (contextual fear conditioning), we examined the ability of animals to remember specific paired-associations between (a) varying flavours of food and (b) the spatial locations in a testing arena where more of each type of flavoured food could be obtained [27]. Such paired-associates (PAs) are discrete memories that may collectively form an organized structure of knowledge called a ‘schema’ [28, 29]. The concept of a ‘schema’ and the likely defining features of such entities have been discussed in recent human neuropsychological literature [30, 31], with our understanding of these being developed further through human analogs of the rodent spatial schema task using functional neuroimaging [32–34]. The aim of the body of work of which this is a part, including these recent human studies, is to secure a better neurobiological understanding of the underlying mechanisms of ‘neural schemas’.

Specifically, the expression patterns of the protein products of the plasticity-related IEGs associated with remembering previously acquired flavour-place PAs and the learning of new ones were compared across four distinct groups of animals that had different experiences on a critical session prior to collection of the brains [14]. Once collected and sectioned, quantitative measurement of EGR-1 and ARC expression was conducted. We predicted that different patterns of IEG products expression would be observed across groups and sought to quantify and characterize their relative activation with regard to how networks of neurons are coordinated during the initial stages of long-term memory formation against a backdrop of a prior knowledge.

## Materials and methods

### Subjects and behavioural procedures

**Fig. 1:**
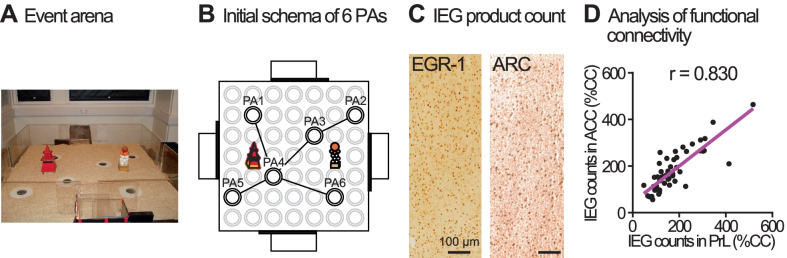
Analysis of functional brain networks. **A** Behavioural equipment called an ‘event arena’ in rat. **B** Behavioural training for an initial schema of 6 flavour-place paired-associates (PAs) in the event arena. **C** Immunohistochemistry for immediate early gene (IEG) products, EGR-1 and ARC, followed by cell counting. **D** Statistical data analysis including inter-regional correlation matrices, hierarchical clustering and network construction. *ACC* anterior cingulate cortex, *CC* caged-control group, *PrL* prelimbic cortex

The subjects (n = 28) were adult male Lister Hooded rats (Charles River), housed in groups and maintained at 90% of their free-feeding weight throughout the experiment. The details of the subjects and apparatus were described fully in our previous paper [14]. The ‘event arena’ was made of transparent Plexiglas (1.6 × 1.6 m), with four adjacent start boxes (Fig. 1A). The floor of the arena, arranged in a 7 × 7 grid of 49 circular holes, was covered with ~ 3 cm of sawdust and had two distinctive intra-arena landmarks (Fig. 1B). The Plexiglas sandwells (6 cm diameter, 5 cm depth) with a metal mesh grid 3.5 cm from the top, in which food rewards (0.5 g pellets manufactured in various flavors, Bio-Serv) could be placed, could be inserted into the circular holes in the floor of the arena. To mask olfactory cues from the rewarded pellets in the sandwell, the sand was mixed with ground pellets powder (25 g powder in 2.5 kg sand). In addition, all sandwells contained 6 g pellets (0.5 g × 2 pellets per flavour) at the bottom of the sandwell that could not be accessed by the animal due to the metal mesh grid. To make food reward available in a sandwell, 3 pellets (0.5 g each) were hidden on the upper surface of the metal mesh grid under the sand layer. The rats could then dig through the sand mixture to search for and retrieve each food pellet.

**Fig. 2:**
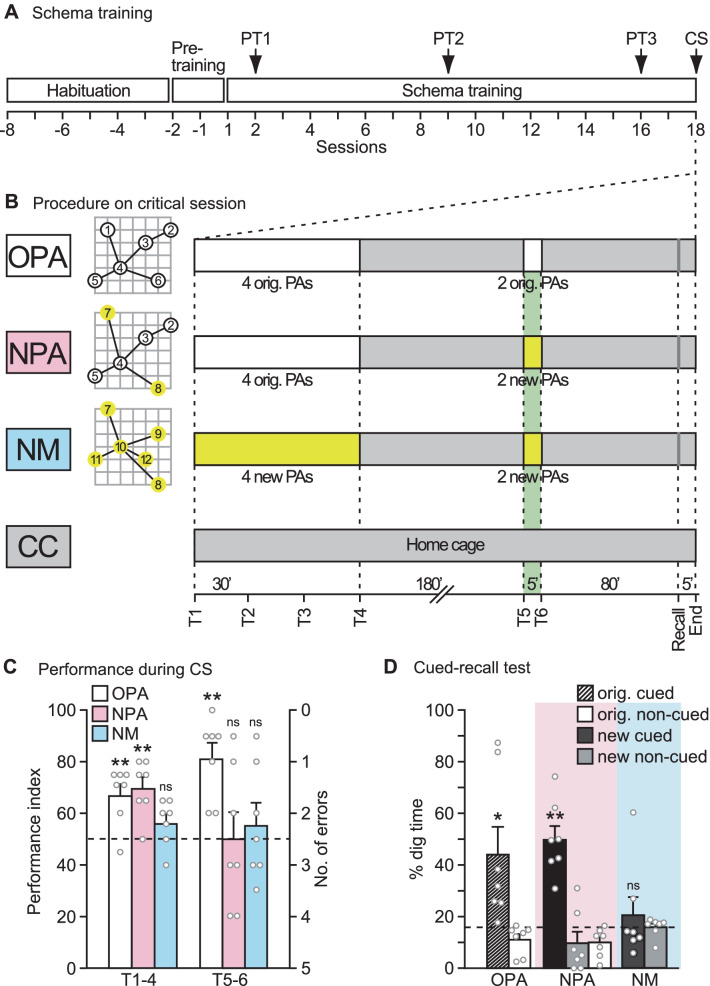
Design for the schema training and the critical session, and performance during critical session. **A** Timeline showing the design of the behavioural experiment including habituation, pre-training, schema training and critical session. PT, probe test; CS, critical session. **B** Timeline showing procedures on the critical session [white boxes, training of original paired-associates (PAs); yellow boxes, training of new PAs; gray boxes, staying in the home cage; green background, trials for which EGR-1 and ARC expression were measured]. The trained animals were divided into three groups in the critical session (Group OPA, original paired-associates, white; Group NPA, new paired-associates, pink; Group NM, new map, blue; n = 7 in each group). A caged-control (CC) group stayed in a home cage throughout the experiment (n = 7). Recall, cued-recall test; T1-6, trials 1–6; End, collecting the brain sample. **C** Performance divided between the first 4 (T1-4) and last 2 trials (T5-6) in Groups OPA (white bars), NPA (pink bars) and NM (blue bars) during critical session. A dashed line, chance level. **D** Cued-recall test 80 min after trial 6. The graph represents percentage dig time at the original cued location (a slash bar), the average of the original non-cued location (white bars), the new cued location (black bars) as well as the average of the new non-cued location (gray bars) in Groups OPA, NPA and NM. A dashed line, chance level. ns, no significance; *p < 0.05; **p < 0.01 versus chance level (t-test). Data are means ± SEM

The behavioural experiment mainly divided into the following phases: habituation, pre-training, original PAs schema training and critical session (Fig. 2A, B). During original PAs schema training, rats were trained to learn six PAs of flavours of food and the locations in the event arena (Fig. 1B). Guided by the retrieval cue of different flavours of food given in one of the start boxes, the animals learned to recall the location of the appropriate sandwell, where they were rewarded by retrieving more of that same flavoured food. The timing of events on the critical session are outlined in Fig. 2B and were also described previously [14].

During each trial, experimenters recorded the number of errors the rats made before approaching the rewarded correct sandwell. Using the number of errors, a performance index score is calculated using 100 − [100 × (errors/5)]—i.e., 50% at 2.5 errors. In the cued-recall test (probe test), all 6 sandwells were open as usual and the rats could dig in any of them, but none contained any accessible food pellets as reward. The rats were cued in the startbox with a single flavor as usual, and then allowed into the arena for a total of 120 s. Dig time at each sandwell was measured semi-automatically using custom built software (developed in LabVIEW, National Instruments). The experimenter recorded the time rats spent digging at each of 6 sandwells, and the relative proportion of time at original cued, new cued, original non-cued and new non-cued sandwells was calculated. All animal experimental procedures were compliant with the United Kingdom Home Office Animal Procedures Act (1986) conducted under a Project Licence (PPL 60/4566).

### Immunohistochemistry and IEG product counts

On the last of the critical session, rats were perfused, the brains rapidly removed on ice, and then sectioned in the coronal plane (40 µm) throughout the full anterior–posterior extent of the brain. Different sections were then subject to immunohistochemical staining using a rabbit anti-EGR-1 antibody (sc-189, 1:3000 dilution; Santa Cruz Biotechnology) or a rabbit anti-ARC antibody (OP-1, 1:2000 dilution [35]). EGR-1 and ARC expressions were analysed in 12 brain regions, namely the prelimbic cortex (PrL), infralimbic cortex (IL), orbito-frontal cortex (Orb), insular cortex (Ins), anterior cingulate cortex (ACC), somatosensory cortex (Ssp), the anterior and posterior retrosplenial cortices (aRC and pRC), the hippocampus [dentate gyrus (DG), CA3 and CA1], and the entorhinal cortex (EC). The details of the immunocytochemical and microscopy procedures were described previously [14].

### Inter-regional correlation matrices

Across 12 brain regions, all possible pairwise correlations between the grouped IEG product counts (EGR-1 and ARC counts) were determined by computing Pearson correlation coefficients. The data were shown as color-coded correlation matrices with MATLAB (Mathworks).

### Hierarchical clustering

**Fig. 3:**
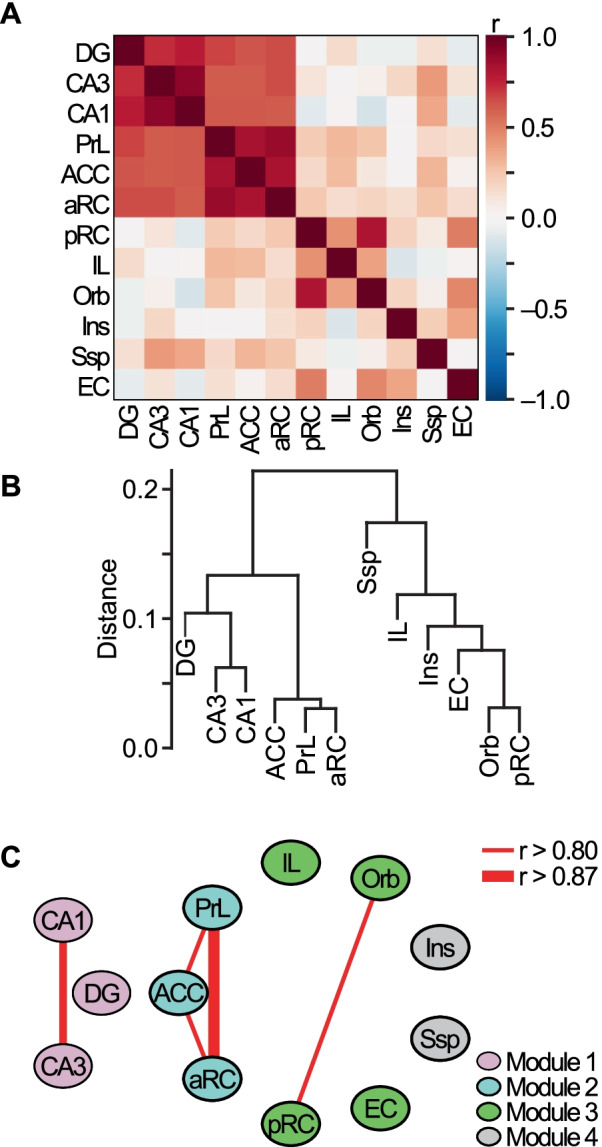
Functional connectivity in association with new memory encoding and/or retrieval of paired-associates. **A** Inter-regional correlation matrix for grouped IEG product counts (EGR-1 and ARC counts) in all 4 experimental groups (Groups OPA, NPA, NM and CC). Colors show correlation strength. Right, color scale. **B** Each brain region was hierarchically clustered by grouped IEG product counts (EGR-1 and ARC counts) in all 4 experimental groups with the Pearson’s correlation serving as the distance measure to represent average linkage. **C** A network graph was generated by connecting each brain region (node) based on the strongest correlations (Pearson’s r > 0.80 or 0.87). Modules were defined by correlation-based cluster analysis with the Louvain method. *ACC* anterior cingulate cortex, *aRC* anterior retrosplenial cortex, *DG* dentate gyrus, *EC* lateral entorhinal cortex, *IL* infralimbic cortex, *Ins* insular cortex, *Orb* orbitofrontal cortex, *pRC* posterior retrosplenial cortex, *PrL* prelimbic cortex, *Ssp* somatosensory cortex

**Fig. 4:**
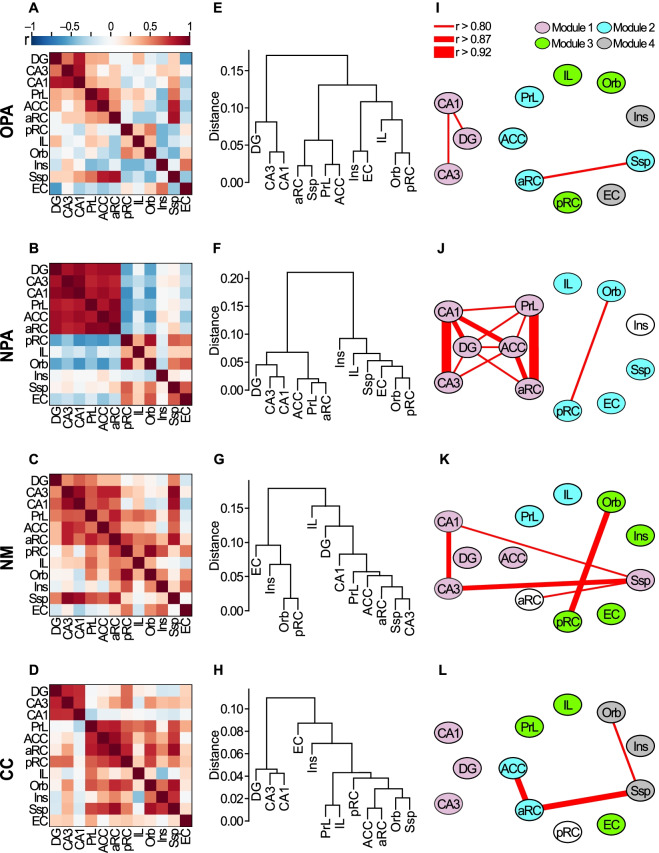
Functional connectivity in the individual groups. **A**–**D** Inter-regional correlation matrices for grouped IEG product counts (EGR-1 and ARC) in Groups OPA (**A)**, NPA (**B)**, NM (**C)** and CC (**D)**. Colors show correlation strength. Top, color scale. **E**–**H** Each brain region was hierarchically clustered by grouped IEG product counts with the Pearson’s correlation as the distance measure and average linkage in Groups OPA (**E)**, NPA (**F)**, NM (**G)** and CC (**H)**. **I**–**L** Network graphs were generated by connecting each brain region (node) based on the strongest correlations (Pearson’s r > 0.80, 0.87 or 0.92) in Groups OPA (**I)**, NPA (**J)**, NM (**K)** and CC (**L)**. Modules were defined by correlation-based cluster analysis with the Louvain method. In Group NPA, hippocampal regions (DG, CA3 and CA1) have strong connections with midline neocortical regions (PrL, ACC and aRC) and these 6 brain regions constitute NPA module 1. *ACC* anterior cingulate cortex, *aRC* anterior retrosplenial cortex, *DG* dentate gyrus, *EC* lateral entorhinal cortex, *IL* infralimbic cortex, *Ins* insular cortex, *Orb* orbitofrontal cortex, *pRC* posterior retrosplenial cortex, *PrL* prelimbic cortex, *Ssp* somatosensory cortex

Average-linkage hierarchical clustering was performed for the 12 brain regions grouped by 4 groups (Fig. 3B) or individual groups (Fig. 4E–H) as described before [36]. Briefly, dissimilarity index (distance) between individual pairs of brain regions was computed based on the Pearson function in “amap” package (http://cran.r-project.org/web/packages/amap/index.html);

$${\text{Distance}} = \frac{{1 - \sum\nolimits_{i} {x_{i} y_{i} } }}{{\sqrt {\sum\nolimits_{i} {x_{i}^{2} \sum\nolimits_{i} y_{i}^{2} } } }}$$

where x and y are IEG product counts in a pair of brain regions. Then, the most similar pair (i.e., the minimum distance) is joined in a dendrogram. This step was repeated until all brain regions are merged. For the dissimilarity index of a merged pair, average-linkage method [37] was used, where the dissimilarity between the merged pair and the others was the average of the pair of dissimilarities in each case. These analyses were performed using R (Ver 2.8.0, http://www.r-project.org/).

### R-value thresholding for network construction

R-value based networks were constructed by thresholding inter-regional correlations in grouped by 4 experimental groups (Fig. 3C) or each of the 4 experimental groups (Fig. 4 I–L) by considering correlations with Pearson’s r > 0.80, > 0.87, or > 0.92 (corresponding to a significance level of p < 0.001, 0.0001, 0.00001, respectively). The nodes represent each brain region and the correlations that survived thresholding were considered ‘connections’.

### Louvain method to detect modules without thresholds

Module detection was carried out using the community_louvain function in the Brain Connectivity Toolbox within MATLAB [38]. This function implements the Louvain method for correlation-based cluster analysis without thresholding [39]. The community_louvain function includes a gamma parameter, which controls the size and number of detected modules. A smaller gamma value provides the detection of a small number of larger modules, while larger gamma values provide the detection of a high number of smaller modules [40]. To find an optimal gamma value, we carried out module detection using a range of values (0.1–2.0, increments of 0.01), and assessed the stability of the resultant modules as described previously [40]. Briefly, we carried out module detection 100 times on the correlation network followed by pairwise comparisons of the similarity of the module clustering between the runs. To assess similarity between two module groupings, we used ami function within MATLAB [41]. We have selecting a largest gamma parameter (i.e., 1.2) which shows the perfect similarity between the 100 runs to obtain the stable module detection results (Additional file 1: Fig. S1).

### Fisher z-transformation

Fisher z-transformation was used to compare the difference between two correlation coefficients. Fisher z-transformation of correlation coefficient is defined as;

$$z=\frac{1}{2}\mathrm{ln}\left(\frac{1+r}{1-r}\right)$$

where *r* is the correlation coefficient. Fisher’s statistic for comparing the correlation coefficients from two samples is as follows;

$$D=\frac{{Z}_{1}-{Z}_{2}}{\sqrt{\frac{1}{{n}_{1}-3}+\frac{1}{{n}_{2}-3}}}$$

where *D* is the normalized differences between correlation coefficient of group1 and group2. *Z*_*t*_ and *n*_*t*_ reprensnt Fisher z-transformation of correlation coefficients and sample numbers of group t, respectively. Then, the *p*-values are computed by treating *D* as a normal random variable.

### Statistical analyses

**Fig. 5:**
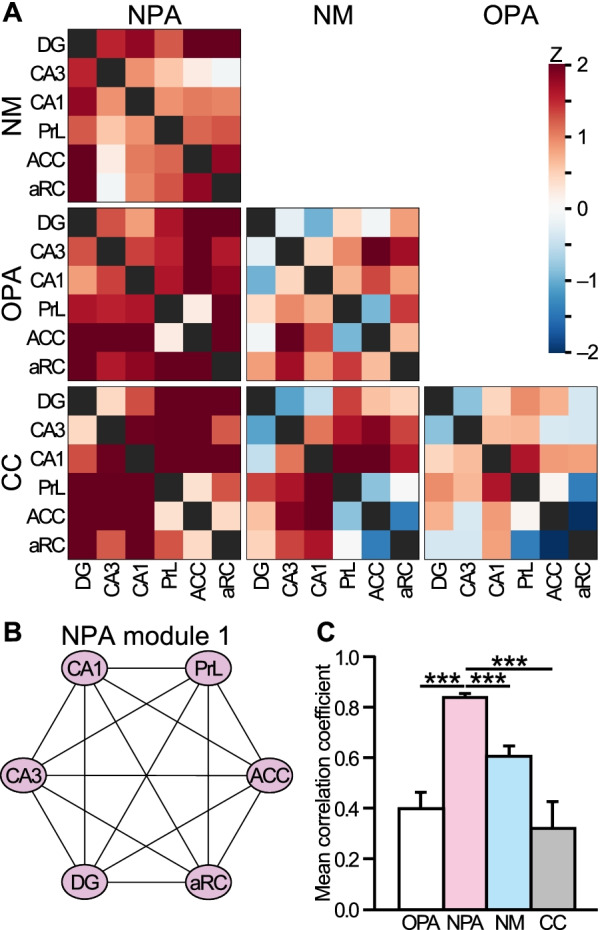
Strong functional connectivity during encoding of new PAs against the backdrop of the relevant schema. **A** The differences between the Fisher z-transformed correlational matrices for the 6 identified brain regions across groups. **B** Six brain regions constituted NPA module 1. **C** Mean correlation coefficients among pairs of regions shown in **B**. Group NPA significantly higher correlation coefficients than any other groups. ***p < 0.0001 (paired t-test with Bonferroni adjustments)

Statistical analyses were performed using SPSS version 19 (IBM). All data are expressed as mean ± SEM. Statistical significance was determined as follows. In behavioural tests (Fig. 2C, D), a one-way ANOVA and one-sample t-test were used. To compare the strength of functional connections (Fig. 5C), a paired t-test with Bonferroni correction was used. All statistical tests were two-tailed.

## Results

### Differential behavioural patterns on the session when expression of immediate early gene product was measured

The training and other aspects of the behaviour of the animals in an “event arena” have been described elsewhere [14]. Briefly, the event arena (Fig. 1A) contained 6 sandwells at various locations, from which food reward could be obtained, that were accessible from any of 4 start boxes at the sides of the arena. An initial schema of 6 flavour-place PAs is shown in Fig. 1B. The timeline of the 18 sessions of training are shown in Fig. 2A. The animals were decapitated 90 min after the start of training of the last two PAs of the critical session (Fig. 2B). The brains were removed and prepared for immunocytochemistry of EGR-1 and ARC. Twelve distinct brain regions (see “Materials and methods”) were identified for quantitative analysis, and EGR-1 and ARC positive cells were counted (Fig. 1C). Our previous analysis of the IEG product expression data indicated differential brain-region specific patterns of EGR-1 and ARC expression across groups, with a focus on the prelimbic cortex (PrL) and hippocampus [14]. Here, the focus of the new analyses is both different and novel. To identify brain region networks underlying successful assimilation of new information into the relevant schema, comparisons were made of the IEG product counts in the full set of pairs of brain regions across all 12 regions by using correlational and clustering analyses (Fig. 1D).

In the behabioural study, it is necessary to describe differential patterns of behaviour among groups that were observed on the critical session during which expression of IEG products was measured (Fig. 2). There were four groups of animals (n = 28) divided into three groups of trained animals (n = 7 in each group) and one caged-control group (*Group CC*; n = 7). The purpose of Group CC was to provide an initial baseline of IEG product expression against which overall changes in the other groups could be observed, with the primary focus of interest in this project being the spatial patterns of IEG product co-expression rather than the overall level of any one IEG product. The experimental groups had 17 sessions of training (Fig. 2A) and then different experiences on the critical session scheduled for session 18. These were: *Group original PA (OPA)* which was required to retrieve previously acquired knowledge—that is to remember each of the six flavour-place PAs that had been trained previously; *Group new PA (NPA)* which was required to remember 4 of the 6 PAs, but then also to learn 2 new PAs and assimilate them into the existing schema; and *Group new map (NM)* which was exposed to an entirely new map involving 6 new PAs with both new flavours and new locations, the learning of which normally takes approximately 15–20 sessions (Fig. 2B; see Tse et al. [14] for fuller details).

The timing of events on the critical session (CS in Fig. 2A) was considered carefully. This session lasted approximately 360 min, with the first 4 trials (trials 1–4) in each animal at 30 min intervals, followed by a delay of 180 min, and then 2 further trials (trials 5 and 6) at an interval of 5 min (Fig. 2B). This was followed by a cued-recall test at 80 min and collection of the brain samples at 85 min. This schedule enabled EGR-1 and ARC expression associated with the last 2 PAs (trials 5 and 6), as measured using immunocytochemistry, would have occurred around 90 min after their training at the point that the brain samples were collected, whereas EGR-1 and ARC expression associated with the first 4 PAs (trials 1–4) would have been beyond the likely peak of expression [42, 43] (Fig. 2B). The cued-recall test was conducted 5 min before decapitation, a procedure that would have had no measureable effect on IEG product expression within such a short time period.

The behavioural results, divided between the first 4 (trials 1–4) and last 2 trials (trials 5 and 6), showed that Groups OPA and NPA were performing at about 70% correct over trials 1–4 (Fig. 2C). For the last 2 trials (trials 5 and 6), Group OPA had only to express their previously acquired knowledge and showed a trend towards even better performance; whereas Group NPA which had to learn 2 new PAs showed chance performance. Performance falling to chance in Group NPA on trials 5 and 6 is fully as expected, for while these animals would have been learning these new PAs, they would not have known which location to visit when confronted by a new flavoured pellet as a ‘cue’ in the start box (see “Materials and methods”). This is a critical point about the experimental design: Group NPA, showing good performance on the first 4 PAs reflected having 'activated' the existing neural schema. The following exposure to the two new flavours in the start box, and the finding of these flavoured foods in neighbouring sandwell locations, would have occurred against the backdrop of the activated schema. That they performed at chance on these last two trials was as expected, but memory encoding of 2 new PAs would have been happening. This was shown in Tse et al*.* (2007) [27] for which post-learning cued-recall test data is presented for the identical condition. Group NM, faced with 6 new PAs throughout both phases of training, performed at chance throughout the critical session because these animals would have been unable to benefit from previously acquired knowledge beyond the general requirements of the task. An ANOVA of these behavioural data showed a significant Groups × Trials interaction reflecting the pattern just described (F_2, 18_ = 4.52, p < 0.05). However, when the animals were given cued-recall test 5 min before collecting the brain samples, Group NPA now showed that the single experience of discovering the new flavoured foods at each of two novel locations in the arena (trials 5 and 6) had been enough for single-trial learning (Fig. 2D). In contrast, Group NM displayed no evidence of successful learning.

Since these data indicate that the groups behaved differently on the 2 last training trials (trials 5 and 6) and cued-recall test in the critical session, EGR-1 and ARC expression triggered by these 2 trials is the focus of the remaining analyses. On these trials, Group OPA was well above chance, while Groups NPA and NM were at chance. However, Group NPA was successful in learning of new PAs that could be encoded and assimilated into the existing schema, and thus we may expect the expression pattern for plasticity-related IEG products of this group to be different from that of the others. Group OPA performed well in the final cued-recall test but would not have had to learn anything new during trials 5 and 6.

### Functional connectivity in association with memory encoding and/or retrieval of prior knowledge

We performed the brain region network analysis using grouped normalized EGR-1 and ARC counts, because EGR-1 and ARC counts significantly correlated in some pairs of brain regions, but not in others (Additional file 1: Fig. S2). Correlational analyses with Pearson’s moment product correlation coefficient were conducted to investigate whether the expression pattern of IEG products in one brain region was associated with that in another brain region in association with new memory encoding and/or retrieval of old prior knowledge. Figure 3A shows a correlation matrix for the 12 brain regions that we analysed with the 2 IEG products counts for whole 4 groups (i.e., Groups OPA, NPA, NM and CC). Warmer colours indicate stronger positive correlation. We found that several pairs of regions showed relatively strong correlation. We considered only the strongest correlations (Pearson’s r > 0.80). One pattern represents the strong correlation between areas CA3 and CA1 of the hippocampal formation (see also Fig. 3C). Another pattern represents an association between certain neocortical structures along the midline of the brain—the prelimbic zone of the medial prefrontal cortex (PrL), the anterior cingulate (ACC), and the anterior region of the retrosplenial cortex (aRC). The third patterm of relatively high association was between the orbitofrontal cortex (Orb) and posterior retrosplenial cortex (pRC).

Using the same data set, we performed hierarchical cluster analysis in which distances were measured with the Pearson function (see “Materials and methods”). This analysis demonstrated that the patterms of relatively high association shown in Fig. 3A are tightly clustered (Fig. 3B).

For identification of modules with correlation-based cluster analysis, the Louvain method was implemented to the correlational matrix. This analysis revealed 4 modules (Fig. 3C). Module 1 included dentate gyrus (DG), CA3 and CA1 of the hippocampal formation. Module 2 included midline neocortical regions (PrL, ACC and aRC). Module 3 included the infralimbic cortex (IL), Orb, pRC and the entorhinal cortex (EC). Module 4 included the insular cortex (Ins) and somatosensory cortex (Ssp).

Separate analyses for EGR-1 (Additional file 1: Fig. S3A, C) or ARC counts (Additional file 1: Fig. S3B, D) did not recapitulate those for combining EGR-1 and ARC counts (Fig. 3).

### Brain region networks associated with new PAs encoding against the background of a relevant schema

We further examined the functional connectivity across the individual groups. Group OPA does not display any strong correlations except for DG-CA1, CA3-CA1 and aRC-Ssp pairs (Fig. 4A, E, I). We identified four “OPA modules” (Fig. 4I). Group NPA, the group that would have successfully assimilated two new PAs into the existing schema, shows a remarkably tight association between the hippocampus (DG, CA3 and CA1) and midline neocortical regions (PrL, ACC and aRC) (Fig. 4B, J). A hierarchical cluster analysis shows that hippocampal and midline neocortical regions clustered together in Group NPA (Fig. 4F). This tight cluster was identified “NPA module 1” (Fig. 4J) and not apparent in any other groups (Fig. 4I, K, L). Group NM shows a much more diffuse pattern (Fig. 4C, G, K), perhaps arising in association with the failure of this group to learn much new information in a single session in which they were presented with 6 new PAs that were unrelated to prior knowledge. Last, Group CC displays a kind of ‘resting state’ connectivity but a clear separation between allo- and neocortical regions (Fig. 4D, H, L).

Our analyses reveal that: (1) successful assimilation of two new PAs into the existing relevant schema involves remarkable interactions between the hippocampus (DG, CA3 and CA1) and midline neocortical regions (PrL, ACC and aRC); and (2) these 6 brain regions were classified into NPA module 1 (Fig. 5B). To assess the strength of functional connections of NPA module 1 in each group, we compared all possible pairwise correlations of the Fisher z-transformed matrices of the 6 identified regions across groups (Fig. 5A). The z-scores of each group and their p values are shown in Additional file 2: Table S1. Groups NPA-OPA, NPA-NM and NPA-CC pairs show relatively bigger diffrences than other group paries.

We further compared the mean correlation coefficients of every pair of the 6 identified regions across groups (Fig. 5C). Group NPA shows a significantly higher mean correlation coefficient compared to those of Groups OPA, NM and CC (paired t-test with Bonferroni correction: Group NPA vs Groups OPA, NM or CC, p < 0.001 in each case).

## Discussion

The key findings of this study are: (1) the correlations of expression of plasticity-related IEG products between brain regions in association with new learning constitute a network of brain regions collectively involved in memory encoding and/or retrieval of PAs; (2) the coordinated network patterns vary as a function of the degree of congruence between the new information and the prior knowledge that the animals had acquired in earlier training; and (3) coordination of the neural network between midline neocortical structures (PrL, ACC and aRC) and hippocampal (DG, CA3 and CA1) regions is the strongest during the assimilation of new associative memories into the relevant schema.

We have previously shown that hippocampal lesions, if made at least 48 h after the assimilation of new PAs, leaves the memory of these new PAs intact. This indicates that activation of a neocortical network is enough for successful memory retrieval. However, such lesions completely disrupt the subsequent memory encoding of new PAs into the schema [27]. Additionally, pharmacological blockade of AMPA (α-amino-3-hydroxy-5-methyl-4-isoxazole propionate)-type glutamate receptor in prefrontal regions (PrL and ACC) immediately before encoding of new PAs inhibits successful learning of new PAs [14, 44]. These data raise the possibility that a coordinated functional network between the hippocampal formation and the neocortex regions *causally* supports the encoding of new PAs against the backdrop of relevant prior knowledge. Indeed, our correlational and clustering analyses (Fig. 4) reveals that hippocampal and midline neocortical regions are closely clustered and constitute one module in Group NPA, but not in the other groups. Group NPA also showed the highest mean correlation coefficient between pairs of midline neocortical-hippocampal networks (Fig. 5). Our previous pharmacological studies also showed an intriguing dissociation between encoding of new PAs and the retrieval of previously acquired knowledge in PrL and ACC. Specifically, blocking of the NMDA (N-methyl-d-aspartate)-type glutamate receptor, which is a key molecule for synaptic plasticity, by AP5 in either PrL or ACC disrupted encoding of new PAs, but had no effect on the retrieval of original schema [14, 44]. These results imply that midline neocortical and hippocampal connectivity based on plasticity-related IEG product mapping in Group NPA might be ‘plasticity-related’ rather than ‘activity-related’ functional connectivity.

A number of studies of IEG activation have examined the relative activation of an IEG in one brain region compared to that in another in a range of different learning and memory tasks—spatial memory, recognition, procedural learning [11]. A key idea to emerge from these pioneering IEG studies, as well as lesion studies, is Aggleton’s concept of an “expanded hippocampal system” in which performance depends on the connectivity of the hippocampal formation to a number of thalamic and neocortical structures. Our findings with paired-associate learning are completely consistent with this general approach. More recent work has also raised critical issues about the nature of the control groups against which IEG expression in a specific training condition should be compared [45]. Shires and Aggleton [45] argued, for example, that we need to dissect the differential contributions of sensory experience, motor activity, stress and other variables beyond ‘cognition’ in assessing different patterns of IEG expression across the brain. Although our study did include Group CC (a caged-control), whose analytic value for assessing expression of IEG product can be questioned [45], it is really the different patterns across the three trained groups upon which our conclusions about extended encoding networks is based. The object of Group CC was precisely to provide a baseline with which to measure and then observe changes seen at the time of memory encoding and/or retrieval of PAs by trained animals.

Our analyses of Group OPA (original paired-associates, no new learning) are, in this respect, similar to those of Wheeler and colleages [19] who identified distinct networks at different time-points after training, excepting that our experiment differs in one very important respect as stressed in the Introduction. Whereas Wheeler and colleages [19] examined time-dependent changes in IEG product expression associated with *memory retrieval*, we looked at patterns of IEG product expression associated with *memory encoding* against the backdrop of the previously learned schema. We also focused on plasticity-related IEG products (EGR-1 and ARC) whereas Wheeler and colleages [19] analysed a neural activity-related IEG product (c-Fos). Interestingly, in another study of context fear conditioning [15], *Fos*-associated channelrhodopsin tagging of neurons was observed in the retrosplenial cortex (RSC) that must have occurred at the time of memory encoding as selective optogenetic activation soon after encoding was successful in eliciting learned behavior. Like Wheeler et al. [19], however, Cowensage et al. [15] indicate that the memory traces in RSC formed at encoding needed to be stabilised over time for natural cues (i.e., the context) to successfully elicit learned behavior in the absence of the hippocampus. A recent study demonstrated that the memory traces in PrL and rostral part of ACC formed during contextual fear conditioning were required for memory retrieval more than 12 days later, that hippocampal activity supported their functional maturation [17]. Our use of extensive previous training and the creation of a schema obviates the necessity for long periods of post-training memory consolidation as assimilation into such a schema appears to take place within 48 h [27].

The “association with” encoding and/or retrieval should not be taken as definitive that the patterns of IEG product expression observed, or the inter-regional conectivity measured, are necessarily *caused* by encoding or by retrieval. The associated pharmacological and optogenetic inhibition studies are suggestive, but there are conceptual issues also to consider. In the earlier study of Wheeler et al. [19], there was no opportunity for new fear learning in the sense that re-exposure to the context was not associated with additional presentations of the unconditional shock stimulus. However, the phenomenon of reconsolidation [46] raises the possibility that a retrieval procedure (re-exposure to the fearful context) could nonetheless be associated with new encoding (or at least updating), and the extent to which this happens could be time-dependent. Likewise, our Group OPA was subject to retrieval and thus the potential to re-encode the previously trained PAs, whereas Group NPA was subject to new learning procedures (i.e., memory encoding) and retrieval (of the schema). Group NPA displayed evidence of new learning of new PAs in a single critical session. Group NM received 6 new PAs and did not show evidence of learning.

Potential limitations of our approach include the followings: First, while the number of brain regions analyzed is relatively low (12 brain regions), we did find midline neocortical-hippocampal connectivity was strongly associated with successful memory encoding of new PAs against the backdrop of the schema. Second, we combined EGR-1 and ARC counts and used them for our brain region network analysis. The combined analysis of EGR-1 and ARC counts (Fig. 3) showed different results from separate analyses of EGR-1 and ARC counts (Additional file 1: Fig. S3), suggesting that the results are a consequence of combining 2 IEG product counts. Since spatio-temporal expression patterns and associated functions are not identical between EGR-1 and ARC [47], we reason that the combined analysis of EGR-1 and ARC counts provides additional insight in functional connectivity that separate analyses cannot. However, the conclusion requires further confirmation with independent samples.

**Fig. 6:**
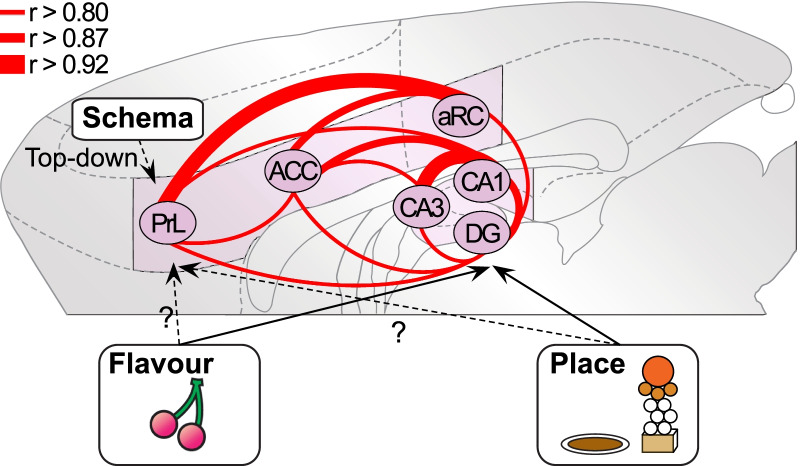
Network model during memory assimilation into schema. The framework emerging from correlational and clustering analyses. Plasticity-related midline neocortical-hippocampal connectivity (Pearson’s r > 0.80, 0.87 or 0.92) is strongly associated with successful memory encoding of new paired-associates against the backdrop of the schema. *ACC* anterior cingulate cortex, *aRC* anterior retrosplenial cortex, *DG* dentate gyrus, *PrL* prelimbic cortex

The devil-is-in-the-detail and these qualifications point to complications in relating patterns of IEG product expression with putative memory-related processes such as encoding or retrieval. However, our findings do indicate that Group NPA was unusual in having demonstrable connectivity between hippocampal and certain midline neocortical structures in circumstances in which new PAs are processed and learned rapidly against the backdrop of relevant and activated prior knowledge. None of the other groups showed comparable patterns of functional connectivity. The patterns we have observed are consistent with an emerging framework (Fig. 6) in which novel information is associated within the hippocampal formation but that the assimilation of new PAs is then differentially assimilated into a neocortical network. For this task, the network involves the prelimbic, anterior cingulate and retrosplenial regions, but of course it will be task-specific. Such a framework is consonant with a study on the human analog of the rodent spatial schema task with neuroimaging studies [33]. They showed that successful encoding of new object-location PAs in the backdrop of the relevant schema was associated with activity in the the ventromedial prefrontal cortex and retrosplenial cortex. Furthermore, the assimilation of new PAs was associated with coupling of the ventromedial prefrontal cortex with the hippocampus and retrosplenial cortex. Aggleton’s concept of an “extended hippocampal system” derived from early observations of IEG activation during different types of learning [11] and complementary ideas in the domain of human memory [48], are converging with the schema studies in rodents and humans onto new ideas about the interconnecting networks mediating memory formation and persistence.

## Conclusion

In this study, we applied correlational and clustering analyses to data on expression of two IEG products that were acquired in one of our previous studies [14]. Our main finding is that midline neocortical-hippocampal connectivity is strongly associated with successful memory encoding of new PAs against the backdrop of the schema, compared to both (1) unsuccessful memory encoding of new PAs that are not relevant to the schema, and (2) the mere retrieval of the previously learned schema. This finding suggests that the certain midline neocortical and hippocampal networks support the assimilation of newly encoded associative memories into a relevant schema.

## Supplementary Information

**Additional file 1: Figure S1.** Mean adjusted mutual information (AMI) as a function of gamma values for the Louvain method. Louvain module detection was performed 100 times for grouped EGR-1 and ARC counts of the whole group (i.e. Groups OPA, NPA, NM and CC) on a range of gamma values. The mean AMI between each pair of the 100 runs at each gamma value was shown. A higher AMI means more stable module detection between runs. The red dotted line shows the value selected for gamma in this study. Mean ± standard deviation. **Figure S2.** Correlational analysis of normalized EGR-1 and ARC counts for all 4 experimental groups (Groups OPA, NPA, NM and CC) in each of the 12 brain regions. ACC, anterior cingulate cortex; aRC, anterior retrosplenial cortex; CC, caged-control group; DG, dentate gyrus; EC, lateral entorhinal cortex; IL, infralimbic cortex; Ins, insular cortex; Orb, orbitofrontal cortex; pRC, posterior retrosplenial cortex; PrL, prelimbic cortex; Ssp, somatosensory cortex. **Figure S3.** Correlational and clustering analyses for EGR-1 and ARC counts. (A and B) Inter-regional correlation matrices for EGR-1 (A) and ARC (B) counts in all 4 experimental groups. Colors show correlation coefficients. (C and D) Network graphs were generated by connecting each brain region (node) based on the strongest correlations (Pearson’s r > 0.80, 0.87 or 0.92) for EGR-1 (C) and ARC (D) counts in all 4 experimental groups. Modules were defined by correlation-based cluster analysis with the Louvain method.

**Additional file 2: Table S1.** The Fisher z-transformed differences between correlation coefficients of each pair of groups (Groups OPA, NPA, NM and CC) and their p values. The Fisher z-values (upper triangular matrix) and their p values (lower triangular matrix) are shown in each pair of groups. ACC, anterior cingulate cortex; aRC, anterior retrosplenial cortex; DG, dentate gyrus; PrL, prelimbic cortex.

## Data Availability

The datasets generated and analyzed during the current study are available from the corresponding authors on reasonable request.

## Abbreviations

ACC: Anterior cingulate cortex
AMPA: α-Amino-3-hydroxy-5-methyl-4-isoxazole propionate
ARC: Activity-regulated cytoskeleton-associated protein
aRC: Anterior retrosplenial cortex
DG: Dentate gyrus
EC: Entorhinal cortex
EGR-1: Early growth response protein 1
IEG: Immediate early gene
IL: Infralimbic cortex
Ins: Insular cortex
NMDA: N-methyl-d-aspartate
Orb: Orbito-frontal cortex
PA: Paired-associate
pRC: Posterior retrosplenial cortex
PrL: Prelimbic cortex
Ssp: Somatosensory cortex

## References

[1] Hebb DO (1949). The organization of behaviour.

[2] Kandel ER (1978). A cell-biological approach to learning.

[3] Martin SJ, Grimwood PD, Morris RGM (2000). Synaptic plasticity and memory: an evaluation of the hypothesis. Annu Rev Neurosci.

[4] Takeuchi T, Duszkiewicz AJ, Morris RGM (2014). The synaptic plasticity and memory hypothesis: encoding, storage and persistence. Philos Trans R Soc B Biol Sci.

[5] McGaugh JL (2000). Memory–a century of consolidation. Science.

[6] Okuda K, Hojgaard K, Privitera L, Bayraktar G, Takeuchi T (2020). Initial memory consolidation and the synaptic tagging and capture hypothesis. Eur J Neurosci.

[7] Frankland PW, Bontempi B (2005). The organization of recent and remote memories. Nat Rev Neurosci.

[8] Wang S-H, Morris RGM (2010). Hippocampal-neocortical interactions in memory formation, consolidation, and reconsolidation. Annu Rev Psychol.

[9] Squire LR, Genzel L, Wixted JT, Morris RGM (2015). Memory consolidation. Cold Spring Harb Perspect Biol.

[10] Squire LR (2004). Memory systems of the brain: a brief history and current perspective. Neurobiol Learn Mem.

[11] Aggleton JP, Pearce JM (2001). Neural systems underlying episodic memory: insights from animal research. Philos Trans R Soc Lond B Biol Sci.

[12] DeNardo LA, Liu CD, Allen WE, Adams EL, Friedmann D, Fu L, Guenthner CJ, Tessier-Lavigne M, Luo L (2019). Temporal evolution of cortical ensembles promoting remote memory retrieval. Nat Neurosci.

[13] Lesburgueres E, Gobbo OL, Alaux-Cantin S, Hambucken A, Trifilieff P, Bontempi B (2011). Early tagging of cortical networks is required for the formation of enduring associative memory. Science.

[14] Tse D, Takeuchi T, Kakeyama M, Kajii Y, Okuno H, Tohyama C, Bito H, Morris RGM (2011). Schema-dependent gene activation and memory encoding in neocortex. Science.

[15] Cowansage KK, Shuman T, Dillingham BC, Chang A, Golshani P, Mayford M (2014). Direct reactivation of a coherent neocortical memory of context. Neuron.

[16] Bero AW, Meng J, Cho S, Shen AH, Canter RG, Ericsson M, Tsai LH (2014). Early remodeling of the neocortex upon episodic memory encoding. Proc Natl Acad Sci U S A.

[17] Kitamura T, Ogawa SK, Roy DS, Okuyama T, Morrissey MD, Smith LM, Redondo RL, Tonegawa S (2017). Engrams and circuits crucial for systems consolidation of a memory. Science.

[18] Hasan M, Kanna MS, Jun W, Ramkrishnan AS, Iqbal Z, Lee Y, Li Y (2019). Schema-like learning and memory consolidation acting through myelination. FASEB J.

[19] Wheeler AL, Teixeira CM, Wang AH, Xiong X, Kovacevic N, Lerch JP, McIntosh AR, Parkinson J, Frankland PW (2013). Identification of a functional connectome for long-term fear memory in mice. PLoS Comput Biol.

[20] Vetere G, Kenney JW, Tran LM, Xia F, Steadman PE, Parkinson J, Josselyn SA, Frankland PW (2017). Chemogenetic interrogation of a brain-wide fear memory network in mice. Neuron.

[21] Tronson NC, Taylor JR (2007). Molecular mechanisms of memory reconsolidation. Nat Rev Neurosci.

[22] Yap EL, Greenberg ME (2018). Activity-regulated transcription: bridging the gap between neural activity and behavior. Neuron.

[23] Rao-Ruiz P, Couey JJ, Marcelo IM, Bouwkamp CG, Slump DE, Matos MR, van der Loo RJ, Martins GJ, van den Hout M, van Ijcken WF (2019). Engram-specific transcriptome profiling of contextual memory consolidation. Nat Commun.

[24] Morgan JI, Curran T (1991). Stimulus-transcription coupling in the nervous system: involvement of the inducible proto-oncogenes fos and jun. Annu Rev Neurosci.

[25] Guzowski JF, Setlow B, Wagner EK, McGaugh JL (2001). Experience-dependent gene expression in the rat hippocampus after spatial learning: a comparison of the immediate-early genes Arc, c-fos, and zif268. J Neurosci.

[26] Jones MW, Errington ML, French PJ, Fine A, Bliss TV, Garel S, Charnay P, Bozon B, Laroche S, Davis S (2001). A requirement for the immediate early gene Zif268 in the expression of late LTP and long-term memories. Nat Neurosci.

[27] Tse D, Langston RF, Kakeyama M, Bethus I, Spooner PA, Wood ER, Witter MP, Morris RGM (2007). Schemas and memory consolidation. Science.

[28] Bartlett FC (1932). Remembering; a study in experimental and social psychology.

[29] Bransford J (1979). Human cognition: learning, understanding, and remembering.

[30] Ghosh VE, Gilboa A (2014). What is a memory schema? A historical perspective on current neuroscience literature. Neuropsychologia.

[31] Gilboa A, Marlatte H (2017). Neurobiology of schemas and schema-mediated memory. Trends Cogn Sci.

[32] van Buuren M, Kroes MC, Wagner IC, Genzel L, Morris RGM, Fernandez G (2014). Initial investigation of the effects of an experimentally learned schema on spatial associative memory in humans. J Neurosci.

[33] Sommer T (2017). The emergence of knowledge and how it supports the memory for novel related information. Cereb Cortex.

[34] Guo D, Yang J (2020). Interplay of the long axis of the hippocampus and ventromedial prefrontal cortex in schema-related memory retrieval. Hippocampus.

[35] Okuno H, Akashi K, Ishii Y, Yagishita-Kyo N, Suzuki K, Nonaka M, Kawashima T, Fujii H, Takemoto-Kimura S, Abe M (2012). Inverse synaptic tagging of inactive synapses via dynamic interaction of Arc/Arg3.1 with CaMKII beta. Cell.

[36] Kang HJ, Kawasawa YI, Cheng F, Zhu Y, Xu XM, Li MF, Sousa AMM, Pletikos M, Meyer KA, Sedmak G (2011). Spatio-temporal transcriptome of the human brain. Nature.

[37] Liu X, Zhu XH, Qiu P, Chen W (2012). A correlation-matrix-based hierarchical clustering method for functional connectivity analysis. J Neurosci Methods.

[38] Rubinov M, Sporns O (2010). Complex network measures of brain connectivity: uses and interpretations. Neuroimage.

[39] Blonde VD, Guillaume J-L, Lambiotte R, Lefebvre E (2008). Fast unfolding of communities in large networks. J Stat Mech Theory Exp.

[40] Jackson MA, Bonder MJ, Kuncheva Z, Zierer J, Fu J, Kurilshikov A, Wijmenga C, Zhernakova A, Bell JT, Spector TD (2018). Detection of stable community structures within gut microbiota co-occurrence networks from different human populations. PeerJ.

[41] Nguyen XV. The Adjusted Mutual Information https://www.mathworks.com/matlabcentral/fileexchange/33144-the-adjusted-mutual-information. MATLAB Central File Exchange. Retrieved January 3, 2022. 2022.

[42] Zangenehpour S, Chaudhuri A (2002). Differential induction and decay curves of c-fos and zif268 revealed through dual activity maps. Brain Res Mol Brain Res.

[43] Ramirez-Amaya V, Vazdarjanova A, Mikhael D, Rosi S, Worley PF, Barnes CA (2005). Spatial exploration-induced Arc mRNA and protein expression: evidence for selective, network-specific reactivation. J Neurosci.

[44] Wang SH, Tse D, Morris RGM (2012). Anterior cingulate cortex in schema assimilation and expression. Learn Memory.

[45] Shires KL, Aggleton JP (2008). Mapping immediate-early gene activity in the rat after place learning in a water-maze: the importance of matched control conditions. Eur J Neurosci.

[46] Nader K, Hardt O (2009). A single standard for memory: the case for reconsolidation. Nat Rev Neurosci.

[47] Lonergan ME, Gafford GM, Jarome TJ, Helmstetter FJ (2010). Time-dependent expression of Arc and zif268 after acquisition of fear conditioning. Neural Plast.

[48] Shohamy D, Turk-Browne NB (2013). Mechanisms for widespread hippocampal involvement in cognition. J Exp Psychol Gen.

