# Supplementary material for: Brain region networks for the assimilation of new associative memory into a schema
Source: Mol Brain. 2022 Mar 24;15:24. doi: 10.1186/s13041-022-00908-9 (PMC8943948; doi:10.1186/s13041-022-00908-9)
Supplement: Supplementary file 1 — Additional file 1: Figure S1. Mean adjusted mutual information (AMI) as a function of gamma values for the Louvain method. Louvain module detection was performed 100 times for grouped EGR-1 and ARC counts of the whole group (i.e. Groups OPA, NPA, NM and CC) on a range of gamma values. The mean AMI between each pair of the 100 runs at each gamma value was shown. A higher AMI means more stable module detection between runs. The red dotted line shows the value selected for gamma in this study. Mean ± standard deviation. Figure S2. Correlational analysis of normalized EGR-1 and ARC counts for all 4 experimental groups (Groups OPA, NPA, NM and CC) in each of the 12 brain regions. ACC, anterior cingulate cortex; aRC, anterior retrosplenial cortex; CC, caged-control group; DG, dentate gyrus; EC, lateral entorhinal cortex; IL, infralimbic cortex; Ins, insular cortex; Orb, orbitofrontal cortex; pRC, posterior retrosplenial cortex; PrL, prelimbic cortex; Ssp, somatosensory cortex. Figure S3. Correlational and clustering analyses for EGR-1 and ARC counts. (A and B) Inter-regional correlation matrices for EGR-1 (A) and ARC (B) counts in all 4 experimental groups. Colors show correlation coefficients. (C and D) Network graphs were generated by connecting each brain region (node) based on the strongest correlations (Pearson’s r > 0.80, 0.87 or 0.92) for EGR-1 (C) and ARC (D) counts in all 4 experimental groups. Modules were defined by correlation-based cluster analysis with the Louvain method. [file 13041_2022_908_MOESM1_ESM.docx]

**
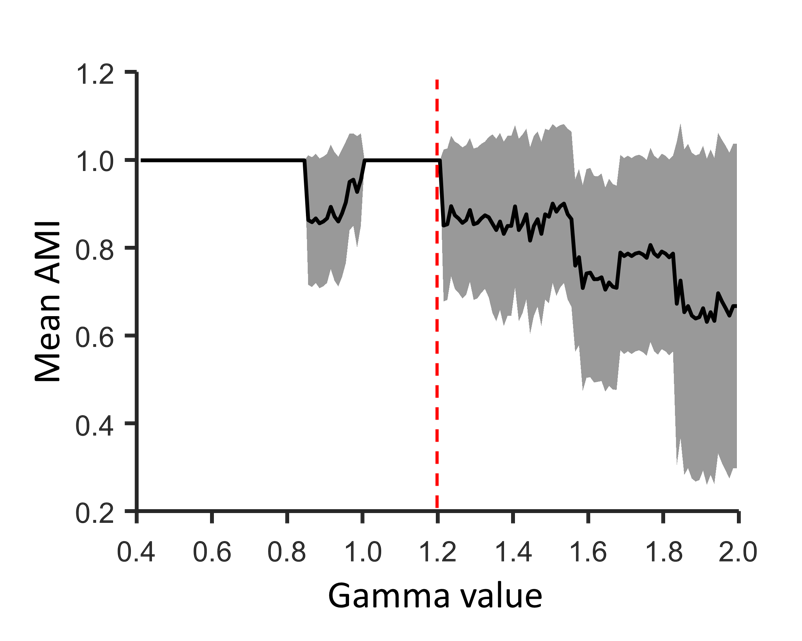
Additional file 1**

**Additional file 1: Figure S1.** Mean adjusted mutual information (AMI) as a function of gamma values for the Louvain method. Louvain module detection was performed 100 times for grouped EGR-1 and ARC counts of the whole group (i.e. Groups OPA, NPA, NM and CC) on a range of gamma values. The mean AMI between each pair of the 100 runs at each gamma value was shown. A higher AMI means more stable module detection between runs. The red dotted line shows the value selected for gamma in this study. Mean ± standard deviation.

**
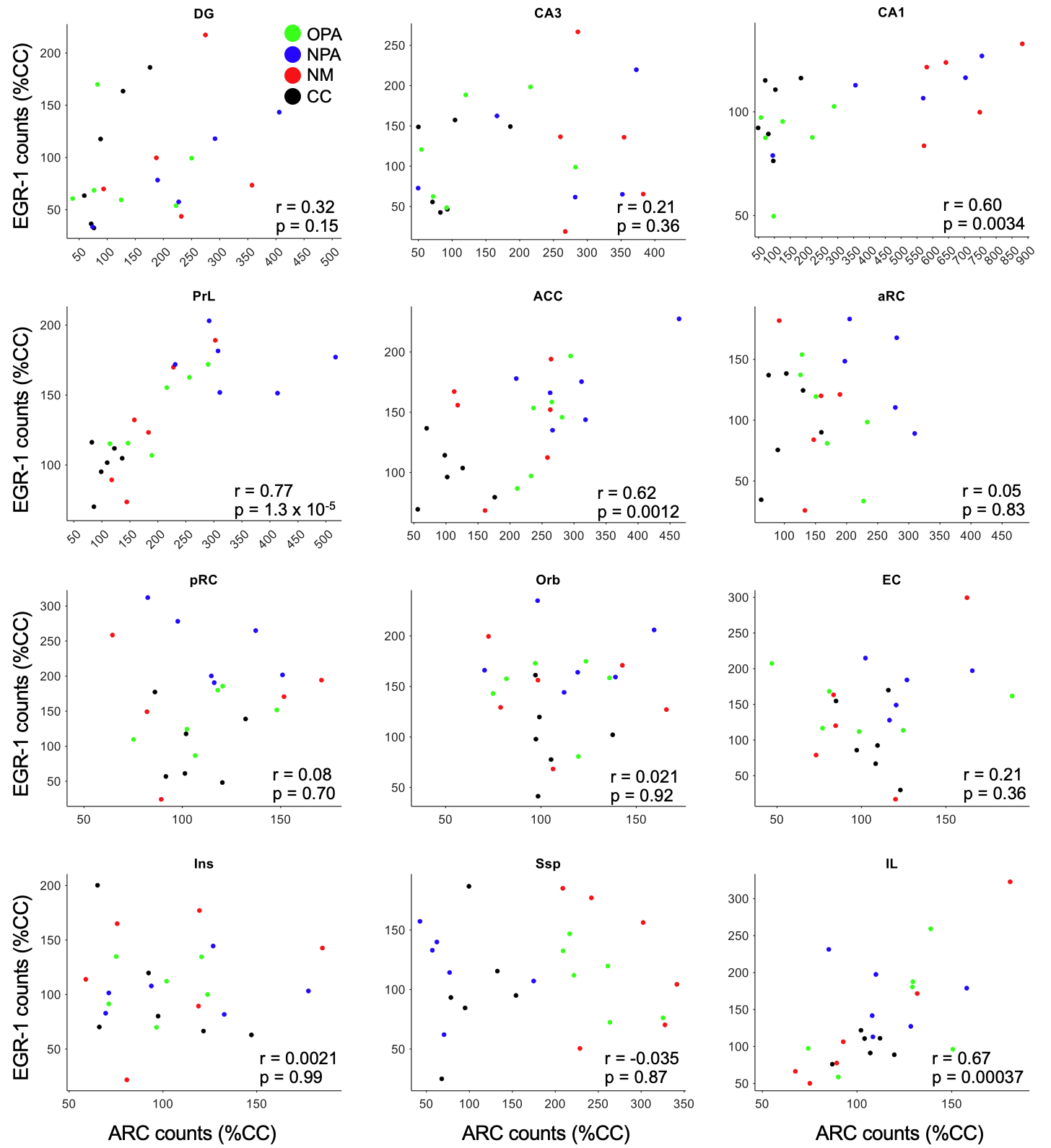
**

**Additional file 1: Figure S2.** Correlational analysis of normalized EGR-1 and ARC counts for all 4 experimental groups (Groups OPA, NPA, NM and CC) in each of the 12 brain regions. ACC, anterior cingulate cortex; aRC, anterior retrosplenial cortex; CC, caged-control group; DG, dentate gyrus; EC, lateral entorhinal cortex; IL, infralimbic cortex; Ins, insular cortex; Orb, orbitofrontal cortex; pRC, posterior retrosplenial cortex; PrL, prelimbic cortex; Ssp, somatosensory cortex.


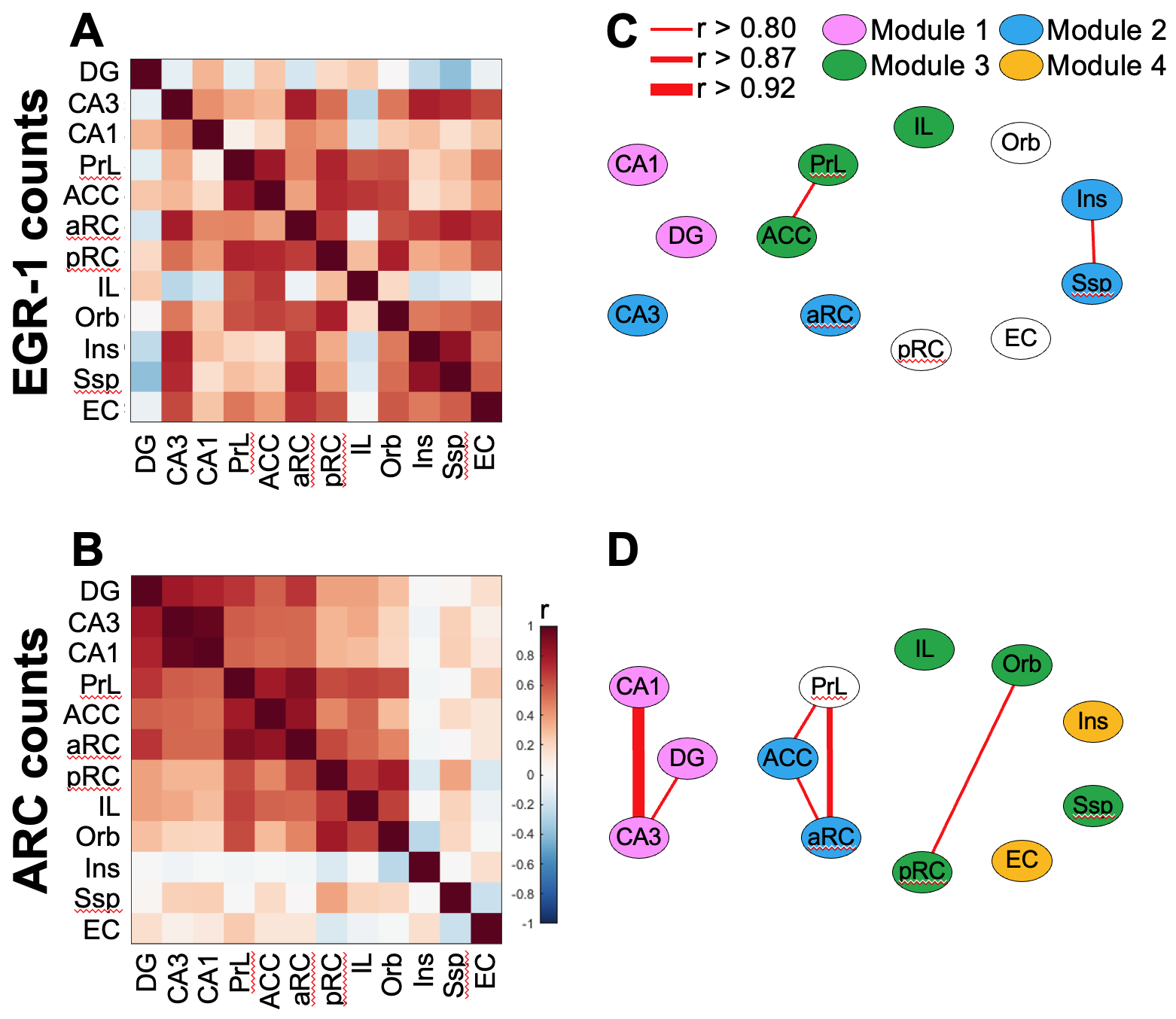


**Additional file 1: Figure S3.** Correlational and clustering analyses for EGR-1 and ARC counts. (**A and B**) Inter-regional correlation matrices for EGR-1 (**A**) and ARC (**B**) counts in all 4 experimental groups. Colors show correlation coefficients. (**C and D**) Network graphs were generated by connecting each brain region (node) based on the strongest correlations (Pearson’s r > 0.80, 0.87 or 0.92) for EGR-1 (**C**) and ARC (**D**) counts in all 4 experimental groups. Modules were defined by correlation-based cluster analysis with the Louvain method.
